# Effect of Etching Procedures on the Adhesion of Biofilm-Coated Dentin

**DOI:** 10.3390/ma13122762

**Published:** 2020-06-18

**Authors:** Bo-Kyung Jeon, Chang-Ha Lee, A Reum Kim, Seung Hyun Han, Hyun-Jung Kim, Sibel A. Antonson, Sun-Young Kim

**Affiliations:** 1 Department of Conservative Dentistry and Dental Research Institute, School of Dentistry, Seoul National University, Seoul 03080, Korea; bk910901@naver.com (B.-K.J.); changha1104@naver.com (C.-H.L.); 2 Department of Oral Microbiology and Immunology, School of Dentistry, Seoul National University, Seoul 08826, Korea; kar1225@naver.com (A.R.K.); shhan-mi@snu.ac.kr (S.H.H.); 3 Department of Conservative Dentistry, Kyung Hee University Dental Hospital, Seoul 02454, Korea; kimhyunjung@khu.ac.kr; 4 Department of Cariology and Restorative Dentistry, College of Dental Medicine, Nova Southeastern University, Fort Lauderdale, FL 33314, USA; Sibel.Antonson@nova.edu

**Keywords:** acid etching, biofilm, confocal laser scanning microscopy, micro-shear bond strength, *S. mutans*

## Abstract

Oral biofilms coat all surfaces in the oral cavity including the exposed dentin surface. This study aimed to investigate biofilm removal by acid etching procedures and the effects of the residual biofilm on dentin surfaces on composite–dentin adhesion. Dentin discs were assigned to five groups: no biofilm formation (C); biofilm formation and no surface treatment (BF); biofilm formation and acid etching (BF-E); biofilm formation and acid etching followed by chlorhexidine soaking (BF-EC); and biofilm formation and rubbing with pumice, followed by acid etching (BF-RE). Biofilms were formed on saliva-precoated dentin discs by soaking the discs in *Streptococcus mutans* (*S. mutans*) suspension. Biofilm removal from the dentin surface was evaluated quantitatively and qualitatively by confocal laser scanning microscopy and scanning electron microscopy, respectively. To compare the bond strength of the biofilm-coated dentin discs with the surface treatments, specimens were assigned to four groups: no biofilm formation and acid etching (C-E); BF-E; BF-EC; and BF-RE. Assessments of the micro-shear bond strength and subsequent failure modes were performed. BF-E and BF-EC did not remove the biofilm, whereas BF-RE partially removed the biofilm attached to the dentin (*p* < 0.05). The bond strength of BF-RE was significantly higher than those of BF-E and BF-EC, but lower than that of C-E (*p* < 0.05). In conclusion, mechanical biofilm removal is recommended before etching procedures to enhance adhesion to the biofilm-coated dentin.

## 1. Introduction

Biofilms coat all surfaces in the oral cavity, including the soft and hard tissues. Oral biofilms on the tooth surface start from the acquired pellicle, which is formed almost instantaneously on all surfaces exposed to oral fluids by the adsorption of salivary proteins [1]. The pellicle provides binding sites for early colonizing bacteria. Microbial adhesion to the pellicle leads to coaggregation and bacterial cell cleavage, and extracellular glucan production is a key component of biofilm formation [2]. This process of biofilm formation through bacterial colonization on dental hard tissues, which is also called dental plaque, plays a key role in the development of caries, gingivitis, and periodontitis [3,4,5]. Oral biofilms also negatively influence the performance of dental restoratives. Biofilm formation is known to deteriorate resin composite and glass-ionomer materials by increasing their surface roughness [6,7] and decreasing their microhardness [8]. The interfacial biofilm also weakens the gap between the tooth and composite resin, leading to the occurrence of secondary caries and eventual pulpal inflammation [9,10,11].

Biofilms on carious or fractured tooth surfaces that are to be restored are generally removed by tooth preparation procedures using a dental bur; therefore, these biofilms may not affect the clinical outcome of resin composite restorations. However, indirect restorations during the temporization period may show a biofilm accumulation on the surface to be bonded. Moreover, non-carious cervical lesions might be affected by the accumulated biofilm on the surface to be bonded with resin composites. Specifically, the cervical region of a tooth, where the oral biofilm is easily formed, is difficult to clean due to anatomical hindrances such as the interproximal and gingival embrasures and the gingival crevices. Moreover, a cervical lesion generally exposes dentin; thus, in addition to adhering to the dentin surface, the biofilm continuously penetrates into the dentinal tubules until it is sealed by a suitable restoration [12]. The oral biofilm on the cervical lesion may interfere with the adhesion of the resin composite restorations, since cavity preparation is seldom performed due to minimally invasive approaches [13]. Although the cleaning of cervical lesions during bonding procedures, i.e., pumice prophylaxis, is recommended for successful resin composite restorations [14], clinicians may neglect this step due to several reasons such as clean-looking surfaces, concerns associated with the time required to clean each tooth, or the possibility of bleeding from a mechanical injury to the gingiva [15]. Clinicians may also assume that the acid etching process would remove the biofilm from the cavity surface based on the conflicting results for the effects of pumice prophylaxis on enamel bonding [15,16,17,18]. However, to the best of our knowledge, the effect of the biofilm removal techniques on the dentin surface and the effect of the residual biofilm on the adhesion of a resin composite to dentin has been rarely studied.

Therefore, the purpose of this study was to investigate the effectiveness of various biofilm removal techniques and identify if any residual biofilm on the dentin surface affects the adhesion between the resin composite and dentin.

## 2. Materials and Methods

### 2.1. Dentin Disc Preparation

Extracted caries-free human third molars were used after receiving approval from the Institutional Review Board of SNUDH (No. CRI085). Teeth were stored in 0.5% chloramin-T solution for disinfection until use. The mid-coronal dentin without pulp tissue was horizontally sectioned with a water-cooled low-speed diamond disc mounted in a sectioning machine (Isomet, Buehler, Lake Bluff, IL, USA). Dentin discs were reduced in thickness on both the pulpal and enamel sides by hand-held grinding with wet 600-grit silicon carbide paper (R&B Co., Daejon, Korea) to reach 600–700 µm in thickness. Disc surfaces were then gradually polished down with 1200-grit silicone-oxide paper (R&B) and examined under a stereomicroscope at 40× magnification (OPMI^®^ pico, Carl Zeiss, Oberkochen, Germany). Dentin discs with pulp horns were discarded. Polished dentin discs were then treated with 17% ethylenediaminetetraacetic acid (EDTA, 99%, Sigma-Aldrich, St. Louis, MO, USA) for 30 s to remove the smear layer. The thickness of the treated dentin discs was 500 ± 80 µm. The dentin discs were sterilized in an autoclave (DAC020, LK Lab, Namyangju, Korea).

### 2.2. Human Saliva Collection and Pre-Coating of Dentin Slices

Saliva for the entire study was obtained from a single 28-year-old healthy volunteer. The saliva was sterilized with a filter system of 0.2-μm pore size (Corning, NY, USA). The autoclaved dentin slices were pre-coated with this sterilized saliva using a dental microbrush 10 times, and then soaked in saliva at 37 °C under 5% CO_2_ aerobic conditions for 24 h before inoculation of the *Streptococcus mutans* (*S. mutans*) solution.

### 2.3. Biofilm Formation

*S. mutans* stock (KCTC3065, KCTC, Jeongeup, Korea) was streaked onto separate blood agar plates (Tryptic Soy agar, Difco, Sparks, MD, USA) containing 2% glucose and 5% sheep blood (BD Biosciences, San Diego, CA, USA), and grown for 48 h. One colony of each bacterial strain was used to inoculate brain heart infusion broth (BHI, BD Biosciences, San Diego, CA, USA) and grown at 37 °C under 5% CO_2_ aerobic conditions for 18 h. Sucrose and BHI were then added to yield an *S. mutans* solution with 1% sucrose and an optical density of 0.2.

Pre-coated dentin discs with saliva were placed in a 12-well plate. Next, 2 mL of BHI media was added to the well of the control group, and 2 mL of the *S. mutans* suspension with 1% sucrose at a final concentration of OD595 = 0.2 (approximately 2.0 × 10^8^ CFU/mL) in BHI media was added to the wells for the experimental groups. The dentin discs were then allowed to form *S. mutans* biofilms on the surface for 72 h at 37 °C under 5% CO_2_ aerobic conditions. The dentin discs were carefully washed twice with phosphate-buffered saline (PBS) to remove the nonattached cells.

### 2.4. Group Assignment and Surface Treatment

A total of 30 dentin discs were randomly assigned to five groups (*n* = 6/group) according to biofilm formation and surface treatment as follows:

(1) Group C (control): no biofilm formation;
(2) Group BF: biofilm formation and no surface treatment;
(3) Group BF-E: biofilm formation and treatment with etching using 37% phosphoric acid (3M ESPE, St. Paul, MN, USA) gel for 15 s and rinsing with distilled water for 30 s;
(4) Group BF-EC: biofilm formation and treatment with etching using 37% phosphoric acid gel for 15 s, soaking in chlorhexidine for 5 min after drying, and rinsing with distilled water for 30 s;
(5) Group BF-RE: biofilm formation and prophylaxis using a rubber cup and plain pumice for 30 s, followed by etching using 37% phosphoric acid gel for 15 s, and rinsing with distilled water for 30 s.

Half of the samples in each group (*n* = 3) were observed with confocal laser scanning microscopy (LSM800, Carl Zeiss, Oberkochen, Germany) and scanning electron microscopy (SEM, S-4700, Hitachi, Tokyo, Japan) to quantitatively and qualitatively assess the biofilm, respectively.

### 2.5. Evaluation of Biofilm with Confocal Laser Scanning Microscopy

The biofilms on the dentin discs were stained using a bacterial viability kit (LIVE/DEAD Baclight Kit, Thermo Fisher Scientific, Waltham, MA, USA). Syto 9 stains all living bacteria in green, and propidium iodide stains dead bacteria in red. After staining, the dentin discs were rinsed with PBS and observed at 10× objective magnification using an LSM800 confocal laser scanning microscopy (CLSM) unit (Carl Zeiss, Oberkochen, Germany). In order to compare the relative volumes of the biofilm formed, a total of five points were designated on the dentin disc: the center point where the long axis and short axis of the dentin disc meet, and points 1 mm apart from the center point at each axis. The fluorescence values of each layer, including living and dead bacteria, were summed up to obtain the relative volume of the biofilm at a given area, which was 638.90 × 638.90 μm^2^ set in 10× magnification of CLSM. The findings at five points were averaged for each group and then compared with each other.

### 2.6. Evaluation of Biofilm with Scanning Electron Microscopy

The remaining half of the biofilm-forming and surface-treated samples (*n* = 3) was prepared for the SEM observation. Attached bacteria were prefixed at 4 °C overnight with PBS containing 2.5% glutaraldehyde (Sigma-Aldrich, St. Louis, MO, USA) and 2% paraformaldehyde (pH 7, Sigma-Aldrich, St. Louis, MO, USA), and then washed with PBS. The samples were subsequently fixed with 1% osmium tetroxide (Sigma-Aldrich, St. Louis, MO, USA) for 1.5 h and then washed three times with distilled water. The samples were dehydrated by replacing the buffer with increasing concentrations of ethanol (Sigma-Aldrich, St. Louis, MO, USA) (70%, 80%, 90%, 95% and 100%, each for 15 min). After drying with hexamethyldisilazane (99.9%, Sigma-Aldrich, St. Louis, MO, USA) and coating with gold sputter, the samples were examined under a SEM (S-4700, Hitachi, Tokyo, Japan).

### 2.7. Specimen Preparation for Bond Strength Test

Sixty extracted caries-free human third molars were used. The teeth were embedded in prefabricated acrylic molds using a self-curing resin. They were mounted and sectioned through the mid-crown using a low-speed diamond disc (Isomet, Buehler, IL, USA) to expose the dentin surface. The exposed dentin surfaces were gradually polished with wet 600-, 800- and 1200-grit silicone-oxide sand papers using a polishing machine (Rotopol-V, Struers, Glasgow, UK). Dentin surfaces were then treated with 17% EDTA for 30 s to remove the smear layer. Specimens were sterilized with an autoclave (DAC020, LK Lab, Namyangju, Korea).

### 2.8. Group Assignment and Surface Treatment for the Bond Strength Test

A biofilm was allowed to form on the dentin surface of a specimen for 72 h in the same manner as described in Section 2.2 and Section 2.3, except that the dentin surface was immersed upside down in a well filled with 1% sucrose *S. mutans* suspension.

A total of 60 specimens were randomly assigned to four groups (*n* = 15/group) according to biofilm formation and surface treatment procedures as follows:

(1) Group C-E (control): no biofilm formation and treatment with etching using 37% phosphoric acid solution for 15 s and rinsing with distilled water for 30 s;
(2) Group BF-E: biofilm formation and treatment with etching using 37% phosphoric acid solution for 15 s and rinsing with distilled water for 30 s;
(3) Group BF-EC: biofilm formation and treatment with etching using 37% phosphoric acid solution for 15 s, soaking into chlorhexidine for 5 min after drying, and rinsing with distilled water for 30 s;
(4) Group BF-RE: Biofilm formation and treatment with rubbing using rubber cup and plain pumice for 30 s, etching using 37% phosphoric acid solution for 15 s, and rinsing with distilled water for 30 s.

### 2.9. Micro-Shear Bond Strength Test and Failure Mode Observation

Dentin surfaces of all specimens were dried for the dentin adhesive application (Single Bond 2; 3M ESPE, St. Paul, MN, USA). The adhesives were applied and light-cured for 10 s with an LED light curing unit (Bluephase 20i, Ivoclar Vivadent, Schaan, Liechtenstein). A polyethylene tube (Tygon E-3603, Scilab Co, Seoul, Korea) of 0.8 mm in diameter and 1 mm in height was used as a mold. The tube was filled with composite resin (Filtek Z-250, 3M ESPE, St. Paul, MN, USA) on the dentin surface and light-cured for 20 s from 1 mm from the top surface of the tube. The intensity of the light curing unit was checked before curing with a calibrated radiometer (Bluephase Meter, Ivoclar Vivadent, Schaan, Liechtenstein) to verify 1200 mW/cm^2^ of output. After light polymerization, the polyethylene tube was removed to leave resin composite cylinders on the dentin surfaces. The specimens were immersed in saline for 24 h at 37 °C.

The micro-shear bond strength test was performed with a universal testing machine (LF Plus; Lloyd Instruments, Fareham, UK). Shear force was applied to the bonding interface using a stainless steel orthodontic wire (0.2 mm in diameter). The wire attached to the load cell was looped around the composite cylinder as close as possible to the bonding interface. The crosshead speed was 0.5 mm/min.

The failure mode was determined by examining the fractured interface of the specimen with a stereoscopic microscope at 40× magnification (Carl Zeiss, Oberkochen, Germany). The failure mode was classified as ‘‘adhesive failure’’ when it occurred between the tooth and the composite resin, and ‘‘mixed failure’’ when both the adhesive failure and cohesive failure within the composite resin occurred simultaneously. When failures occurred within the composite or teeth, they were classified as “cohesive failure in composite” or “cohesive failure in dentin,” respectively.

### 2.10. Statistical Analysis

The remaining biofilm volume per unit area of the dentin surface and the bond strength were analyzed via a one-way analysis of variance (ANOVA). Differences among the groups were assessed via Tukey’s multiple comparison test. The level of significance was set at α = 0.05. All statistical analyses were conducted with GraphPad Prism (Version 8.3.0, GraphPad Software, San Diego, CA, USA)

## 3. Results

### 3.1. Evaluation of Remaining Biofilm on Dentin Surface after Surface Treatment

**Figure 1:**
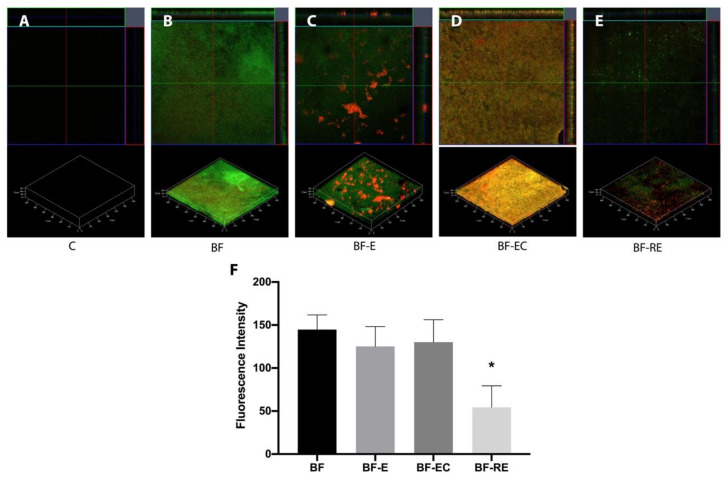
Confocal laser scanning microscopy (CLSM) images of *Streptococcus mutans* (*S. mutans*) biofilm grown on dentin discs after surface treatment (green and red staining represent live and dead bacterial cells, respectively). (**A**) Control, (**B**) biofilm formation and no surface treatment, (**C**) biofilm formation and treatment with acid etching, (**D**) biofilm formation and treatment with acid etching and chlorhexidine, (**E**) biofilm formation and treatment with pumice prophylaxis and acid etching, and (**F**) fluorescence intensity of the different experimental groups. The asterisk (*) indicates statistically significant differences between the groups (*p* < 0.05).

Figure 1 shows the CLSM findings for the remaining biofilm on the dentin surface with different surface treatments. Acid etching (BF-E) caused some dead bacterial cells, and chlorhexidine treatment (BF-EC) increased the dead cells on the dentin surface. However, the total fluorescence intensity, which indicated the biofilm volume, showed no significant difference among groups BF-E, BF-EC, and BF. Prophylaxis with pumice before the acid etching procedure (BF-RE) significantly decreased the biofilm volume on the dentin surface compared with the other groups (*p* < 0.05) (Figure 1F).

**Figure 2:**
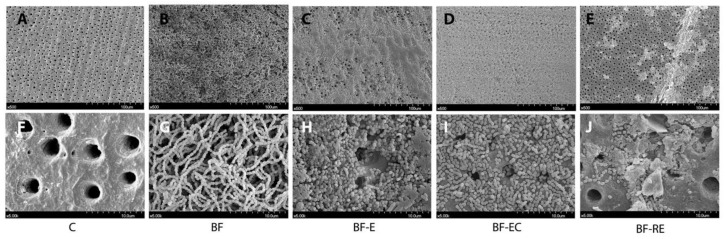
SEM images of *S. mutans* biofilms grown on dentin discs after surface treatment. (**A**,**F**) Control, (**B**,**G**) biofilm formation and no surface treatment, (**C**,**H**) biofilm formation and treatment with acid etching, (**D**,**I**) biofilm formation and treatment with acid etching and chlorhexidine, and (**E**,**J**) biofilm formation and treatment with prophylaxis with pumice and acid etching (**A**–**E**: 500× magnification, **F**–**J**: 5000× magnification).

Figure 2 shows the representative SEM images for the remaining biofilm on the dentin surfaces after different surface treatments. Acid etching either with or without chlorhexidine treatment (BF-E and BF-EC) led to morphological changes in *S. mutans*, including the destruction of the chain structure that was typically observed in the BF group. However, the remaining spherical-shaped bacteria were still partially blocking the dentinal tubules in the BF-E and BF-EC groups. Group BF-RE showed many open dentinal tubules compared with groups of BF-E and BF-EC, but showed some debris, and the remaining spherical-shaped bacteria partially occluded the dentinal tubules.

### 3.2. Evaluation of Bond Strength

**Figure 3:**
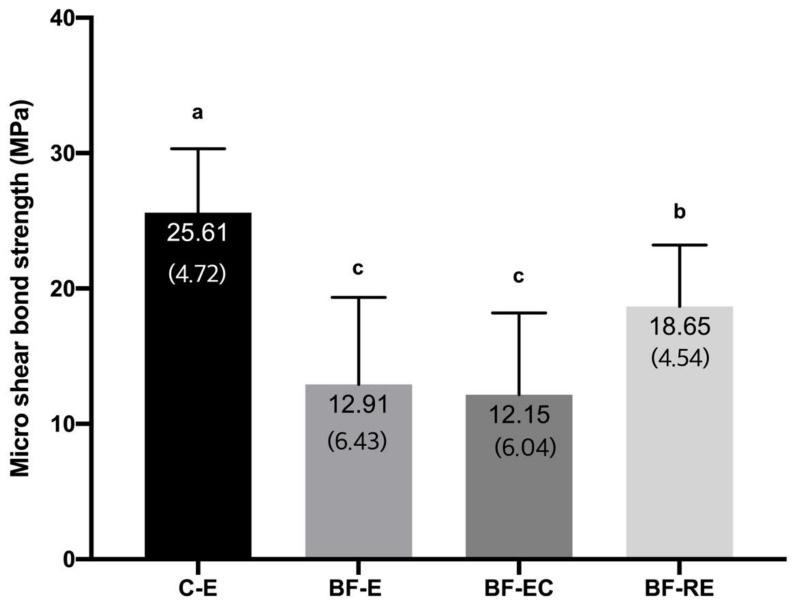
Micro-shear bond strength of the experimental groups (C-E: no biofilm formation and treatment with acid etching, BF-E: biofilm formation and treatment with acid etching, BF-EC: biofilm formation and treatment with acid etching and chlorhexidine, and BF-RE: biofilm formation and treatment with pumice rubbing and acid etching). Numbers in parentheses represent standard deviation values. Different letters (a, b, c) on top of the bar mean statistically significant differences between groups (*p* < 0.05).

Figure 3 shows the micro-shear bond strength values of the composite to biofilm-coated dentin with different surface treatments. BF-E (12.91 ± 6.43 MPa) and BF-EC (12.15 ± 6.04 MPa) showed the lowest bond strength, and the control group (C-E), which had no biofilm coating, presented the highest bond strength (25.61 ± 4.72 MPa) (*p* < 0.05). The bond strength of BF-RE (18.65 ± 4.54 MPa) was significantly higher than that of BF-E and BF-EC but lower than that of C-E (*p* < 0.05).

**Figure 4:**
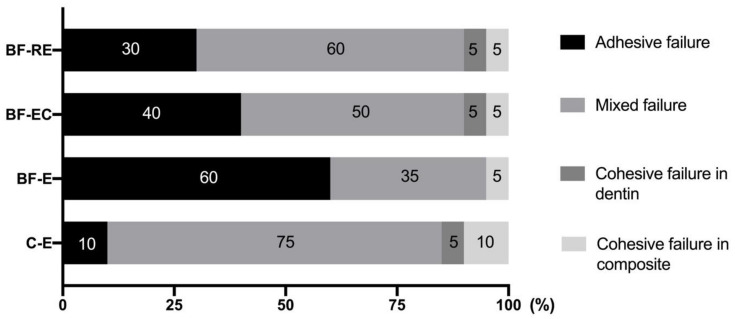
Failure mode analysis of the different experimental groups (C-E: no biofilm formation and treatment with acid etching, BF-E: biofilm formation and treatment with acid etching, BF-EC: biofilm formation and treatment with acid etching and chlorhexidine, and BF-RE: biofilm formation and treatment with prophylaxis with pumice and acid etching). Numbers within each bar indicate the percentage of the corresponding failure mode.

The distribution of the failure modes after the bond strength test is shown in Figure 4. Mixed failure was mainly observed in C-E and BF-RE, and more adhesive failure modes were exhibited in BF-E, BF-EC, and BF-RE compared with C-E.

## 4. Discussion

Acid etching on an adherend substrate is a critical process to achieve successful adhesion between dental hard tissues (i.e., enamel or dentin) and restorative materials [19]. Although the importance of phosphoric acid etching for dentin has been deemphasized due to the development of self-etch adhesives [20] and self-adhesive resin cements [21], selective enamel etching with phosphoric acid is advocated to achieve better clinical performance with these self-etching materials [22]. Since the biofilm coats all surfaces in the oral cavity, the dentin surface to be restored with the composite resin may also be coated for short or long periods. If phosphoric acid etching can effectively remove the biofilm from the surface to which restorations will be bonded, clinicians will be able to obtain a clean and fresh surface predictably and quickly without additional treatment. Unfortunately, this study indicated that phosphoric acid etching either with or without chlorhexidine had effective bactericidal action, but both treatments were unable to completely remove alive and dead bacteria attached to the dentin surface (Figure 1 and Figure 2). This deficiency resulted in significantly lower bond strengths compared with the biofilm-free control group (Figure 3). On the other hand, prophylaxis with a rubber cup and pumice removed the biofilm to a significant level, even though some bacterial cells were still partially covering the dentin surface and were entrapped in the dentinal tubules (Figure 1 and Figure 2). Surface prophylaxis with a rubber cup and pumice before acid etching led to a significantly higher bond strength of the resin composite to the dentin than the rest of the test groups. However, it did not reach to the level of bond strength in the control group, which contained a biofilm-free dentin surface. The failure mode analysis exhibited that the control group without a biofilm formation showed mostly mixed failure and fewer adhesive failures, and groups with a biofilm formation showed more adhesive failures (Figure 4). Adhesive failures indicate unsuccessful integration between the materials, a finding that also supports the lower bond strength found in the biofilm-coated dentin surfaces. These results suggest that biofilms on dentin surfaces cannot be removed by phosphoric acid treatment alone, and that the presence of biofilms on dentin surfaces interferes with the dentin–resin composite adhesion. In addition, this study showed that the adhesion of a biofilm-coated dentin is improved to a certain level through mechanical biofilm removal procedures, such as prophylaxis with pumice.

The attachment of biofilms is known to be related to the roughness and hydrophilicity of the surface, surface energy, and extracellular polymeric substances of the biofilm [23,24,25]. Specifically, the extracellular polymeric substance—a biopolymer of microbial origin consisting of proteins, glycoproteins, and glycolipids—provides functional and structural integrity for biofilms [26,27]. The firm attachment by the extracellular polymeric substance might be the main reason why phosphoric acid etching with or without chlorhexidine could not remove the biofilm from the dentin surface. The remnant biofilm on the dentin surface probably decreased the bond strength (Figure 3). Demineralization of the dentin surface with phosphoric acid in the bonding procedure generally exposes the collagen fibers in the dentin and opens the dentinal tubules, leading to the preparation for micromechanical interlocking with adhesive agents [19]. In the region where the biofilm remains, the dentin surface could not be properly demineralized by phosphoric acid, preventing the appropriate hybridization with collagen fibers and adhesives as well as a resin tag formation within the dentinal tubules [19,28,29]. In the present study, even the mechanical pressure and friction with a rubber cup and pumice did not completely remove the biofilm, and could not restore the bond strength to the level of the biofilm-free group. The time for prophylaxis with pumice might have been insufficient to remove the whole biofilm from the dentin surface in this study. In addition, bacteria being pushed into the dentinal tubules and collagen fibers by pumice prophylaxis might have hindered the resin tag formation through the dentinal tubules and collagen fibers, resulting in a diminished bond strength.

Adhesion between the dentin wall of tooth preparations and resin composites is a critical factor determining the success of direct or indirect restorations using resin composite [19]. Based on the results of this study, efforts to remove the biofilm are essential because the remnant biofilm on the dentin surface hinders the adhesion with the resin composite. To date, no study has attempted to determine the effect of surface treatments for biofilm-coated dentin such as acid etching on the biofilm removal and subsequent adhesion to a resin composite. Several studies have investigated whether pumice prophylaxis of the enamel surface before acid etching affects the adhesion of orthodontic brackets or resin composites [15,17,18]. Most of them have reported that pumice prophylaxis before acid etching had little effect on the enamel bond strength, despite the presence of organic debris on the surface without pumice prophylaxis. The contrary outcomes from this study might be due to differences in the experimental setup of the presence or absence of a biofilm coating, as well as the histological differences in enamel versus dentin. In fact, the biofilm on enamel surfaces can be easily removed via frequent tooth brushing. Additionally, enamel has a smoother and denser surface structure, which makes it more resistant to a biofilm accumulation compared with dentin [30].

As for the removal of surface contaminants on dentin to optimize the adhesion, a number of studies have investigated the effects of several surface treatments, including pumice and chlorhexidine prophylaxis, on the bond strength of dentin to a resin composite cement, although most of the contaminants were not biofilms, but smear debris and remnants of provisional cement. Mechanical prophylaxis using a slurry of pumice and a rubber cup to clean the dental plaque and surface debris is a common procedure for restorative treatment in dentistry. However, the effect of pumice prophylaxis on the bond strength in indirect restorations had shown more or less conflicting results. Some studies reported an increased bond strength of dentin to the resin composite cement by effectively eliminating the remnants of the provisional resin cement [31,32], while other investigations presented no significant differences in the bond strength from the control group where the contaminant was either remnant temporary cement or smear debris [33,34].

Chlorhexidine has been used to clean the preparation surface due to its antibacterial effect, and it can induce durable resin–dentin adhesion by protecting against collagen degradation [35]. The chlorhexidine molecule with a positive charge interacts with the negatively charged substance of the bacterial cell wall when low concentrations are used. The binding of chlorhexidine to the bacterial cell wall changes the osmotic equilibrium of the cell, causing the low-molecular weight substances to leak out. In high concentrations, chlorhexidine penetrates the bacterial cell wall, leading to bacterial cytoplasm precipitation [36,37]. In fact, the bactericidal effect of chlorhexidine was evidenced by a prominent increase in the population of bacterial dead cells in the chlorhexidine-treated groups compared with the group that did not receive chlorhexidine treatment in this study (Figure 1C,D). However, other than the antibacterial effect, chlorhexidine treatment appears to have little ability in removing contaminants, including smear debris and remnants of provisional cement, from the dentin surface [38,39]. As for the biofilm, chlorhexidine treatment could not remove the biofilm in this study, leading to a lower bond strength of the resin composite to the dentin.

Biofilm formation in the oral cavity begins with the colonization of bacteria binding to the receptor structure of the pellicle. With a continuous supply of saliva and sucrose, the biofilm mass on the tooth surface increases [1,3]. In this study, a single species of *S. mutans* was used, and saliva was initially coated but not continuously supplied. Therefore, the appearance of the biofilm may be different from the actual biofilm in the oral cavity, and the binding force between the bacteria and dentin may also be different. In situ experimental setups in the oral cavity might be needed to simulate the actual biofilm coating on tooth surfaces in future studies.

## 5. Conclusions

Based on the results of this study, the biofilm coating on dentin was not removed by 37% phosphoric acid etching with or without chlorhexidine, resulting in a lower bond strength of the resin composite to the dentin. Pumice prophylaxis did not completely remove the biofilm from the dentin surface either, but improved the adhesion of the biofilm-coated dentin. Clinically, the mechanical removal of the biofilm is recommended before etching procedures to enhance the adhesion of biofilm-coated dentin because acid etching alone cannot remove the biofilm from the dentin surface.
